# Tissue-resident macrophages maintain choroidal homeostasis by complement dependent and independent mechanisms

**DOI:** 10.1186/s12974-026-03872-6

**Published:** 2026-05-18

**Authors:** Joyce Gong, Sanaz Ghotbaldini, Ritvik Viniak, Amrita Rajesh, Jeremy A. Lavine

**Affiliations:** Department of Ophthalmology, Feinberg School of Medicine, Northwestern University, 240. E. Huron St., McGaw M343, Chicago, IL 60614 USA

**Keywords:** Age-related macular degeneration (AMD), Alternative complement pathway, Choroidal macrophage, Complement, Tissue-resident macrophage

## Abstract

**Background:**

Age-related macular degeneration (AMD) is characterized by disruption of the choriocapillaris (CC) and retinal pigment epithelium (RPE) dysfunction, leading to drusen accumulation. The CC and RPE form a tightly interdependent unit that maintains homeostasis where the CC supplies oxygen and nutrients to the RPE, while the RPE produces vascular endothelial growth factor (VEGF) to maintain the CC. Genetic studies link alternative complement pathway variants to AMD, and complement deposition on the CC increases during both aging and AMD. Macrophages express complement protein, receptors, and inhibitors, suggesting that they may be a missing link in understanding the role of complement in AMD. In support, previous groups have shown that macrophage depletion disrupts RPE-CC homeostasis, leading to AMD-like pathology, but the mechanism remains unclear.

**Methods and results:**

To investigate the role of macrophages in CC–RPE homeostasis, we generated *Cx3cr1*^*CreER*^*Csf1r*^*i−DTR*^ and *Ms4a3*^*Cre*^*Rosa26*^*DTR*^ mice. In *Cx3cr1*^*CreER*^*Csf1r*^*i−DTR*^ mice, tamoxifen administration induced diphtheria toxin receptor (DTR) expression, allowing ablation of all macrophages (many tamoxifen injections) or long-lived, tissue-resident macrophages (tamoxifen followed by a 3–4 week wash out period). *Ms4a3*^*Cre*^*Rosa26*^*DTR*^ mice were used to deplete monocyte-derived macrophages. Ablation of all macrophages caused decreased CC density, increased CC apoptosis, RPE disorganization, and membrane attack complex (MAC) accumulation. Tissue-resident macrophage ablation phenocopied this result while monocyte-derived macrophage ablation had no phenotype. Additionally, long-term depletion of tissue-resident macrophages led to formation of drusen-like sub-RPE deposits and retinal thinning, mimicking AMD pathology. Finally, pharmacologic depletion of all macrophages similarly reduced CC density to genetic ablation, but *C3*^*−/−*^ mice showed an attenuated phenotype.

**Conclusions:**

These data demonstrate that long-lived, tissue-resident macrophages are essential for maintaining CC–RPE homeostasis while monocyte-derived macrophages are dispensable in this context. Further, ocular macrophage ablation led to MAC accumulation, while *C3*^*−/−*^ mice were resistant to CC regression. Together, these findings suggest that tissue-resident choroidal macrophages maintain CC-RPE homeostasis partially by complement dependent mechanisms. Further, the loss of tissue-resident choroidal macrophages over time is a potential mechanism of AMD pathogenesis.

**Supplementary Information:**

The online version contains supplementary material available at 10.1186/s12974-026-03872-6.

## Background

Age-related macular degeneration (AMD) is the leading cause of blindness in developed countries [1]. Vision loss occurs during advanced AMD, which includes both atrophic and neovascular AMD subtypes [2]. Atrophic AMD is characterized by loss of the retinal pigment epithelium (RPE) and photoreceptors, while neovascular AMD is defined by destructive angiogenesis that leads to rapid and severe vision loss, pathologically termed choroidal neovascularization (CNV) [3, 4].Early and intermediate stage AMD, which only causes mild vision impairment, is pathologically defined by drusen formation, which clinically manifests as small yellow deposits beneath the RPE [5]. Drusen are inflammatory lipoprotein deposits that include vitamin A by-products, complement proteins, amyloid, and lipids [6]. Since a key role of the RPE is the phagocytosis of photoreceptor outer segments, drusen are considered a sign of RPE dysfunction, which is a hallmark of AMD pathogenesis [7]. However, longitudinal imaging studies show that deficits in the choriocapillaris (CC), which is the blood supply to the RPE, predict drusen development and disease progression [8, 9]. The RPE and CC function as a tightly interdependent unit. The RPE secretes trophic factors, most importantly vascular endothelial growth factor (VEGF), which is essential for maintaining CC endothelial cell survival and fenestration [10], while the CC supplies the RPE with oxygen and nutrients that are essential for RPE function. Thus, disruption of this reciprocal relationship is a central feature of AMD pathogenesis [6].

Genome-wide association studies link complement to AMD susceptibility, suggesting that complement overactivation is a key disruptor of the RPE-CC homeostatic unit. Polymorphisms in the complement inhibitors factor H [11, 12] and factor I [13], and activators factor B [14], factor D [15], component 2 [14], and component 3 (C3) [16] have led to in-depth investigations of the role of the alternative complement pathway in AMD pathogenesis. Complement activation causes the release of anaphylatoxins like C3a and C5a, which are chemotactic, and ultimately accumulation of the membrane attack complex (MAC), which causes cell lysis [17]. Importantly, MAC accumulates on the CC with age and further during AMD [18], suggesting that polymorphisms in the alternative complement pathway lead to overactivation and death of the CC and RPE. In support, C3 and C5 complement inhibitors are FDA-approved treatments for advanced atrophic AMD [19, 20], which is characterized by photoreceptor, CC, and RPE death. However, the exact mechanisms by which complement becomes overactivated during AMD and how complement overactivation leads to AMD require further investigation.

Choroidal macrophages are a potential missing link in the RPE-CC homeostatic loop and in understanding the mechanism of complement overactivation during AMD. Choroidal macrophages are present at the level of the CC and increase during AMD [21]. Macrophages are known to express complement components like C3 and C5, complement receptors for anaphylatoxins (C3aR, C5aR), and complement receptors for opsonization (CD11b/CR3, CD11c/CR4), suggesting that choroidal macrophages are a potential key contributor to complement activity in the choroid and CC-RPE homeostasis [22, 23]. In support, ocular macrophage ablation via colony-stimulating factor 1 receptor (CSF1R) inhibition resulted in a significant reduction in CC vascular coverage, accompanied by disruption of the RPE [24]. However, CSF1R inhibition is not fully specific, as it also depletes microglia [25] and can inhibit VEGF receptor 2 (VEGFR2), complicating attribution of these effects to choroidal macrophage loss alone. In a separate study, targeted ablation of FOLR2⁺/LYVE1⁺ choroidal macrophages using *Lyve1*^*Cre*^*Csf1r*^*i−DTR*^ mice treated with topical diphtheria toxin (DT) resulted in CC vascular regression and choroidal thinning [26]. Although this mouse system should not target microglia, microglia were not investigated, and the mechanism of how choroidal macrophage ablation leads to CC-RPE homeostasis disruption and an AMD-like phenotype remains unknown.

To address this mechanistic gap, we investigated 4 mouse models: CSF1R antagonism and genetic ablation of all macrophages, long-lived macrophages, and monocyte-derived macrophages. We found that genetic ablation of all macrophages and long-lived macrophages leads to CC-RPE disruption with MAC accumulation on the CC and an AMD-like phenotype. Further, genetic ablation of monocyte-derived macrophages had no effect on CC-RPE health. Finally, CSF1R antagonism caused CC ablation in wildtype mice with partial rescue in *C3*^*−/−*^ mice. These data demonstrate that long-lived choroidal macrophages maintain CC-RPE homeostasis partially by ultimately preventing complement overactivation; however, whether this is a direct or indirect effect requires further study.

## Methods

### Sex as a biological variable

All studies were carried out on male and female mouse populations. All data were investigated for sex-specific effects, and none were found.

### Experimental animals

Wild-type C57BL/6J (#000664), *Csf1r*^*CAG − LSL−DTR*^ (#0124046), *Cx3cr1*^*CreER*^ (#020940), *C3*^*-/-*^ (#029661), *Ms4a3*^*Cre*^ (#036382), *Rosa26*^*CAG-LSL-DTR*^ (#007900) mice were obtained from the Jackson Laboratory (Bar Harbor, ME, USA). *Cx3cr1*^*CreER*^ and *Csf1r*^*CAG − LSL−DTR*^ were crossed to generate *Cx3cr1*^*CreER*^*Csf1r*^*i-DTR*^ mice. Genotyping was performed by Transnetyx (Cordova, TN, USA) to confirm the presence of the *i-DTR* allele. *Ms4a3*^*Cre*^ and *Rosa26*^*DTR*^ mice were bred to create *Ms4a3*^*Cre*^*Rosa26*^*DTR*^ mice. Mice were bred in-house and maintained in a pathogen-free barrier facility within Northwestern University’s Center for Comparative Medicine (Chicago, IL, USA). All experiments were conducted in accordance with the ARVO Statement for the Use of Animals in Ophthalmic and Vision Research and were approved by the Northwestern University Institutional Animal Care and Use Committee.

### Administration of substances

Tamoxifen (20 mg/ml; T5648, Sigma-Aldrich, St. Louis, MO, USA) was dissolved in corn oil (C8267; Sigma-Aldrich) with shaking at 260 RPM, 37 °C for 2 h. Fresh Tamoxifen solutions were prepared weekly and stored in the dark at room temperature for a maximum of one week. Tamoxifen was given intraperitoneally as indicated by the figure schematics at 100 mg/kg.

Mice received daily intraperitoneal injection of sterile PBS vehicle or 500 ng DT (322326; Sigma-Aldrich, St. Louis, MO, USA) in 0.1 ml PBS as indicated by each figure schematic.

Ki20227, CSF1R Inhibitor, was purchased from Tocris Biosciences (#44-815-0, Bristol, UK) and/or Med Chem Express (HY-10408, Deer Park, NJ, USA). Mice received intraperitoneal injections of DMSO or Ki20227 at 100 mg/kg three times per week for 3 weeks.

### Immunofluorescence imaging of retinal and choroidal flatmounts

Eyes were enucleated, and retinas and choroids were dissected in Tris-buffered saline solution (TBS, #BP24711, Thermo Fisher Scientific, Waltham, MA, USA). Tissues were fixed in 4% paraformaldehyde (15,713-S; Electron Microscopy Sciences, Hatfield, PA, USA) for one hour at room temperature and then washed in TBS. Next, tissues were blocked overnight at 4 °C in the blocking solution consisting of TBS supplemented with 5% donkey serum (S30; Sigma-Aldrich, St. Louis, MO), 2.5% bovine serum albumin (A2153; Sigma-Aldrich), and 0.5% Triton X-100 (X100; Sigma-Aldrich). Primary antibody incubations were performed overnight at 4 °C (see Table 1 for antibodies and dilutions). At this point, the choroid and retinal tissues were processed differently.

**Table 1:** Antibodies used in this study

Antibody	Fluorophore	Manufacturer, Product No., Dilution	Usage
Rat anti-mouse podocalyxin	ࣧ	R&D Systems, MAB1556, 1:200	Primary Ab
Goat anti-mouse podocalyxin	ࣧ	R&D Systems, AF1556, 1:250	Primary Ab
Goat anti-mouse CD31	ࣧ	R&D Systems, AF3628, 1:250	Primary Ab
Rat anti-mouse CD31	ࣧ	Novus Bio, NB600-1475, 1:500	Primary Ab
Goat anti-mouse IBA-1	ࣧ	WAKO, 011-27991, 1:250	Primary Ab
Rabbit anti-mouse IBA-1	ࣧ	WAKO, 016-20001, 1:500	Primary Ab
Rabbit anti-mouse CC3	ࣧ	Cell Signaling, CST9661, 1:400	Primary Ab
Rabbit anti-mouse MAC (C5-9b)	ࣧ	Bioss USA, bs-2673R, 1:200	Primary Ab
Rat anti-mouse CD206	ࣧ	Bio-Rad, MCA2235, 1:200	Primary Ab
Rabbit IgG control	ࣧ	Abcam, ab37415, 1:500	Primary Ab
Rat IgG2b control	ࣧ	Bioxcell, BE0090, 1:500	Primary Ab
Goat IgG control	ࣧ	Invitrogen, 31,245, 1:500	Primary Ab
Donkey Anti-rat	Alexa Fluor 488	ThermoFisher, A21208, 1:500	Secondary Ab
Donkey Anti-rat	Alexa Fluor 555	ThermoFisher, A78945, 1:500	Secondary Ab
Donkey anti-goat	Alexa Fluor 488	Thermo Fisher, A11055, 1:500	Secondary Ab
Donkey anti-goat	Alexa Fluor 555	Thermo Fisher, A21432, 1:500	Secondary Ab
Donkey anti-rabbit	Alexa Fluor 647	Thermo Fisher, A31573, 1:500	Secondary Ab
Phalloidin	Alexa Fluor 405	Thermo Fisher, A30104, 1:500	Secondary Ab
Phalloidin	Alexa Fluor 555	Thermo Fisher, A34055, 1:100	Secondary Ab

Choroids were washed twice in TBS-T (TBS with 0.5% Tween-20 [#00777; Amresco, Solon, OH, USA]), fixed in 4% paraformaldehyde for 1 h, and bleached with 2% hydrogen peroxide (BP2633500; Thermo Fisher Scientific, Waltham, MA, USA) in PBS for 4.5 h at 55 °C. Next, choroids were washed three times in TBS-T, blocked in blocking solution for 30 min at room temperature, and secondary antibody incubations (Table 1) were performed overnight at 4 °C.

Retinas were washed six times in TBS-T and incubated with secondary antibodies overnight at 4 °C (Table 1). The next day, choroidal and retinal tissues were washed six times in TBS-T, followed by mounting on HistoBond microscope slides (16004–406; VWR, Batavia, IL, USA) with Immu-Mount (9990402; Thermo Fisher Scientific). Slides were imaged using a Nikon SoRa Spinning Disk Microscope and Nikon NIS Elements software (Nikon, Melville, NY, USA) at the Northwestern University Center for Advanced Microscopy. Rabbit, rat, and goat isotype IgG control staining was used to ensure staining specificity (Fig S1).

### Image analysis

To ensure unbiased quantification, animal identities were masked for all image analyses. For Figs. 4, 5, and 7, quantification was performed independently by at least two graders. If in agreement, the numbers were averaged. If disagreement was identified, a third reviewer graded the image in a masked fashion and the two nearest neighbors were averaged. The CC area was assessed using Fiji (ImageJ; NIH, Bethesda, MD, USA) with the Labkit plugin. Segmentation of vasculature from background was performed using Labkit’s automated classifier, retrained roughly ten times to optimize reliability and accuracy. Next, binary segmentation masks were imported into Fiji for analysis, and the CC area was calculated with the ‘Analyze Particles’ function. The percent area coverage was determined by dividing the segmented area by the total image area ×100, providing a quantitative metric of vascular density.

RPE density was assessed by first cropping a 650 × 650-pixel region. Individual RPE cell boundaries were then manually traced in Fiji using the polygon selection tool. Next, cell outlines were added to the ROI Manager for area measurement, and RPE density was computed as the total number of traced cells divided by the sum of all measured cell areas. Quantification of the RPE size distribution was done by binning RPE cell area into predefined size categories. For each bin, the number of RPE cells was quantified, and the percentage of cells in each bin was computed by dividing the number of cells in the bin by the total number of RPE cells analyzed.

MAC was quantified in Fiji by equalizing color balance settings across images and then measuring raw fluorescence intensity from a single z-stack slice at the level of the choriocapillaris.

### VEGF ELISA

Experiments were performed as described previously [27], with minor modifications outlined below. Following dissection of eyes in cold PBS, the posterior eye cup, comprising the retina, RPE, choroid, and sclera were isolated. Posterior eye cups from each mouse were pooled to generate a single biological sample. VEGF levels in homogenized sample supernatants were quantified using a Quantikine ELISA kit (MMV00; R&D Systems, Minneapolis, MN, USA) according to the manufacturer’s protocol. Absorbance was measured on a Bio-Rad plate reader (Bio-Rad, Hercules, CA, USA), and VEGF concentrations (pg / ml / mouse) were calculated from standard curves using linear regression analysis in Prism (GraphPad Software LLC, Boston, MA, USA).

### Complement activity ELISA

To assess systemic complement activation, serum was collected via cardiac puncture at the time of sacrifice. Classical complement pathway activity was measured using an ELISA kit (HIT420, Hycult Biotech, Uden, Netherlands) according to the manufacturer’s instructions. Absorbance was measured at 450 nm, and complement activity was expressed as a ratio normalized to a reference serum sample with intact C5b-9 (100% complement activity).

### *In vivo* Optical Coherence Tomography (OCT) imaging

Mice were anesthetized and provided pain prophylaxis as previously described [28]. Eyes were lubricated with 2.5% Hypromellose (Vista Gonio; Hi-Health, City, Country). Mice were next positioned on the Spectralis OCT2 system (Heidelberg Engineering, Germany) for OCT imaging as previously described [29]. High-resolution OCT images of the optic nerve were acquired using the 30× objective, centered on the optic nerve. OCT imaging was performed at baseline, at 1 week, and at 2 weeks after initiation of PBS or DT treatment. The retinal thickness was measured along a line perpendicular to the horizontal axis of the eye, 5.0 a.u. (347 μm) from the optic nerve head. Measurements were initially recorded in arbitrary units and converted to µm² using a scaling factor of 69.44 μm per a.u., based on a known distance in the OCT image. Retinal thickness was defined as the distance from the inner limiting membrane to the top of the RPE.

### Statistics

All data are presented as mean ± SEM. Comparisons between two groups were performed using unpaired two-tailed Student’s t-tests or Welch’s t-test when data were normally distributed. For non-parametric data, Mann–Whitney tests were used. One-way ANOVA followed by Sidak’s multiple comparisons test was used for analyses involving more than two groups. RPE cell size distributions and retinal thickness measurements across conditions and time points were analyzed using two-way ANOVA with Šidák’s multiple comparisons test. Sample sizes for each analysis are indicated in the corresponding figure legends. Statistical significance was defined as *p* < 0.05.

## Results

**Fig. 1:**
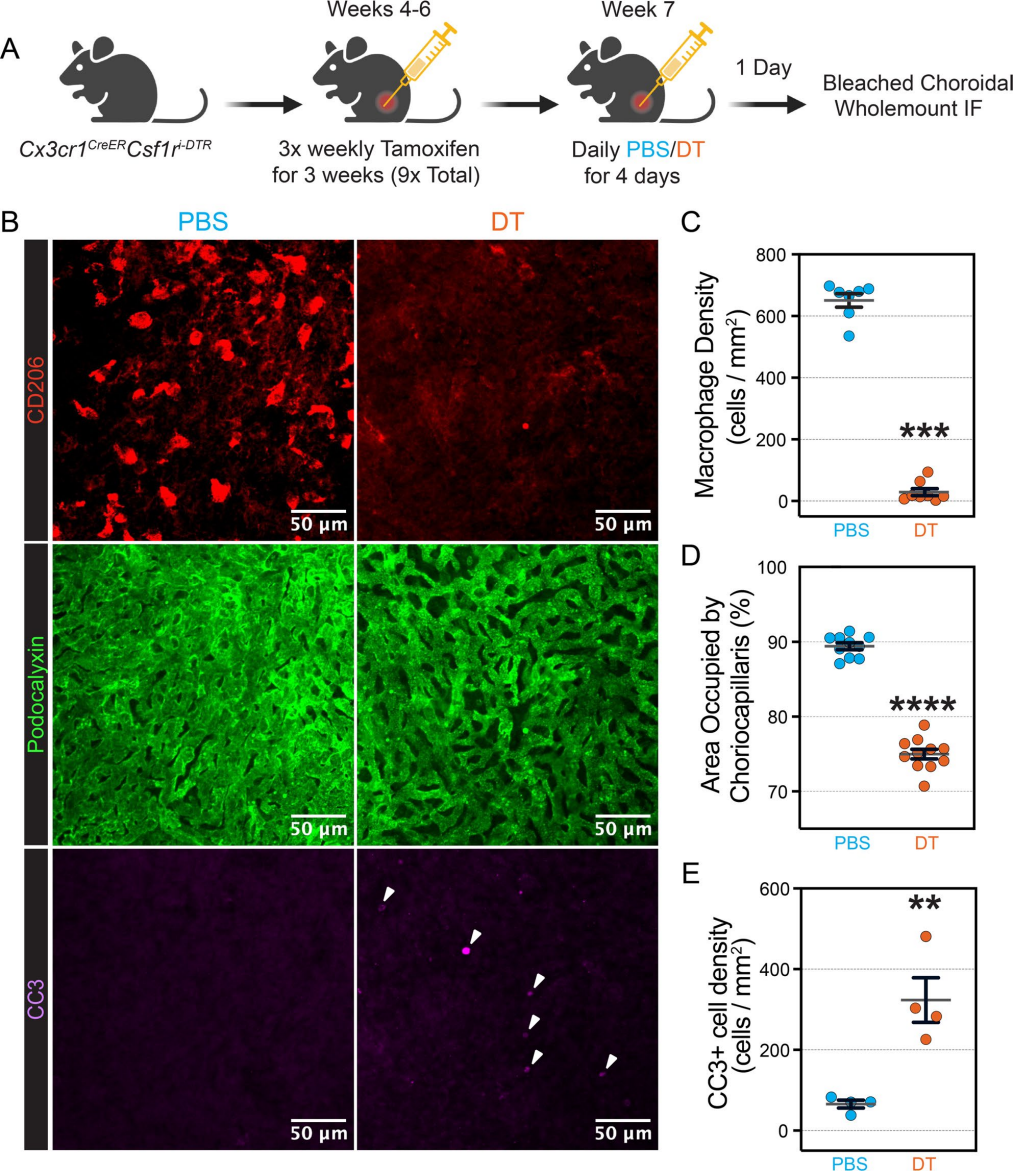
DT treatment in *Cx3cr1*^*CreER*^*Csf1r*^*i-DTR*^ mice reduces macrophage density and causes CC loss. (**A**) Schematic overview of pan-macrophage depletion strategy. (**B**) Representative immunofluorescence images of choroidal wholemounts from PBS vehicle-treated controls and diphtheria toxin (DT)-treated mice, stained for podocalyxin (CC), CD206 (macrophages), and cleaved caspase 3 (CC3; apoptosis marker), with white wedges indicating CC3-positive cells. **C-E**. DT-treated mice exhibited a significant reduction in CC density (Student’s unpaired t-test, *N* = 10–11 mice per group) and macrophage density (Mann–Whitney test, *N* = 7–8 mice per group), accompanied by an increase in CC apoptosis (Welch’s t-test, *N* = 4 mice per group). ** *p* < 0.01, *** *p* < 0.001, **** *p* < 0.0001

To investigate the role of macrophages in choroidal homeostasis, we generated *Cx3cr1*^*CreER*^*Csf1r*^*i−DTR*^ mice to enable inducible macrophage depletion. Mice received 9 doses of tamoxifen over 3 weeks to induce diphtheria toxin receptor (DTR) expression in both tissue resident and monocyte-derived ocular macrophages (Fig. 1A). Next, mice received 4 daily intraperitoneal (IP) injections of either PBS (control) or diphtheria toxin (DT). One day after the final injection, eyes were enucleated for choroidal wholemount immunofluorescence (IF) analysis. We previously showed that this experimental strategy significantly reduced both retinal microglia and choroidal macrophage numbers by > 95% [25]. Representative choroidal wholemount images are shown in Fig. 1B. DT treatment achieved a 95.5% depletion of choroidal macrophages compared to controls (*p* < 0.0001, Fig. 1C). Macrophage ablation resulted in a 14.4% absolute reduction in CC density (*p* < 0.001, Fig. 1D) with a 4.9-fold increase in apoptotic CC cells (*p* < 0.01, Fig. 1E). These data demonstrate that ocular macrophage ablation results in CC degeneration.

**Fig. 2:**
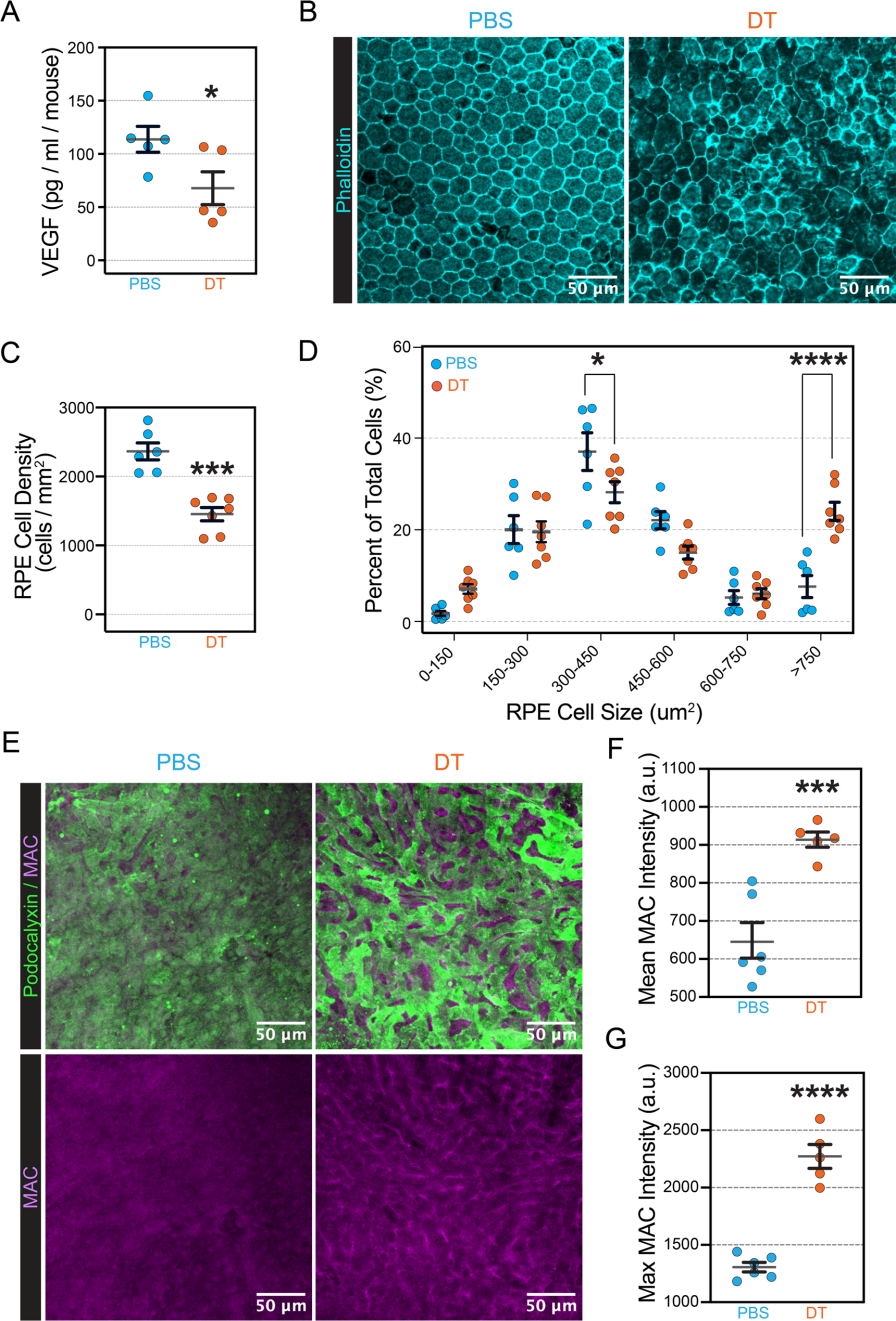
Pan-macrophage ablation disrupts RPE organization and increases MAC deposition. (**A**) Quantification of VEGF expression levels by ELISA (Student’s unpaired t-test, *N* = 5 mice per group). (**B**) Representative choroidal wholemount images of PBS- and DT-treated mice stained with phalloidin (RPE). **C-D**. DT decreased RPE density (Student’s unpaired t-test) and disrupted the uniform organization of the RPE (two-way ANOVA followed by Sidak’s multiple comparisons test, *N* = 6–7 mice per group). **E**. Representative choroidal wholemounts of PBS- and DT-treated mice stained with podocalyxin (CC) and MAC. **F-G**. DT treatment increased mean (**F**) and max (**G**) MAC deposition (Student’s unpaired t-test, *N* = 5–6 mice per group). * *p* < 0.05, *** *p* < 0.001, **** *p* < 0.0001

Since the RPE secretes VEGF to maintain the CC [30], we investigated posterior eye cup VEGF levels after ocular macrophage ablation by ELISA. DT treatment reduced VEGF levels by 40.4% compared to controls (*p* < 0.05, Fig. 2A). Additionally, DT-treated mice exhibited disrupted RPE organization, characterized by a 38.5% reduction in RPE density (*p* < 0.001, Fig. 2B-C) and a 3.2-fold increase in the proportion of enlarged (> 750 µm^2^) cells (*p* < 0.0001, Fig. 2D). These data suggest that ocular macrophage ablation impacts both RPE organization and function, in addition to CC density. Since altered CC-RPE homeostasis is a central pathophysiology of AMD, the alternative complement system is strongly linked to AMD pathogenesis [31, 32], and complement overactivation is postulated to cause CC degeneration during AMD [18], we investigated the terminal product of complement activation, the membrane attack complex (MAC). Macrophage ablation increased mean and maximum MAC levels by 1.4- (*p* < 0.001) and 1.7-fold (*p* < 0.0001), respectively (Fig. 2E-G). In addition, we performed a complement activity ELISA from serum of PBS- and DT-treated mice. We found that DT treatment caused a 2-fold reduction in serum complement activity (*p* < 0.01, Fig S2). These findings indicate that ocular macrophages are essential for maintaining CC-RPE homeostasis, potentially by inhibiting complement overactivation.

**Fig. 3:**
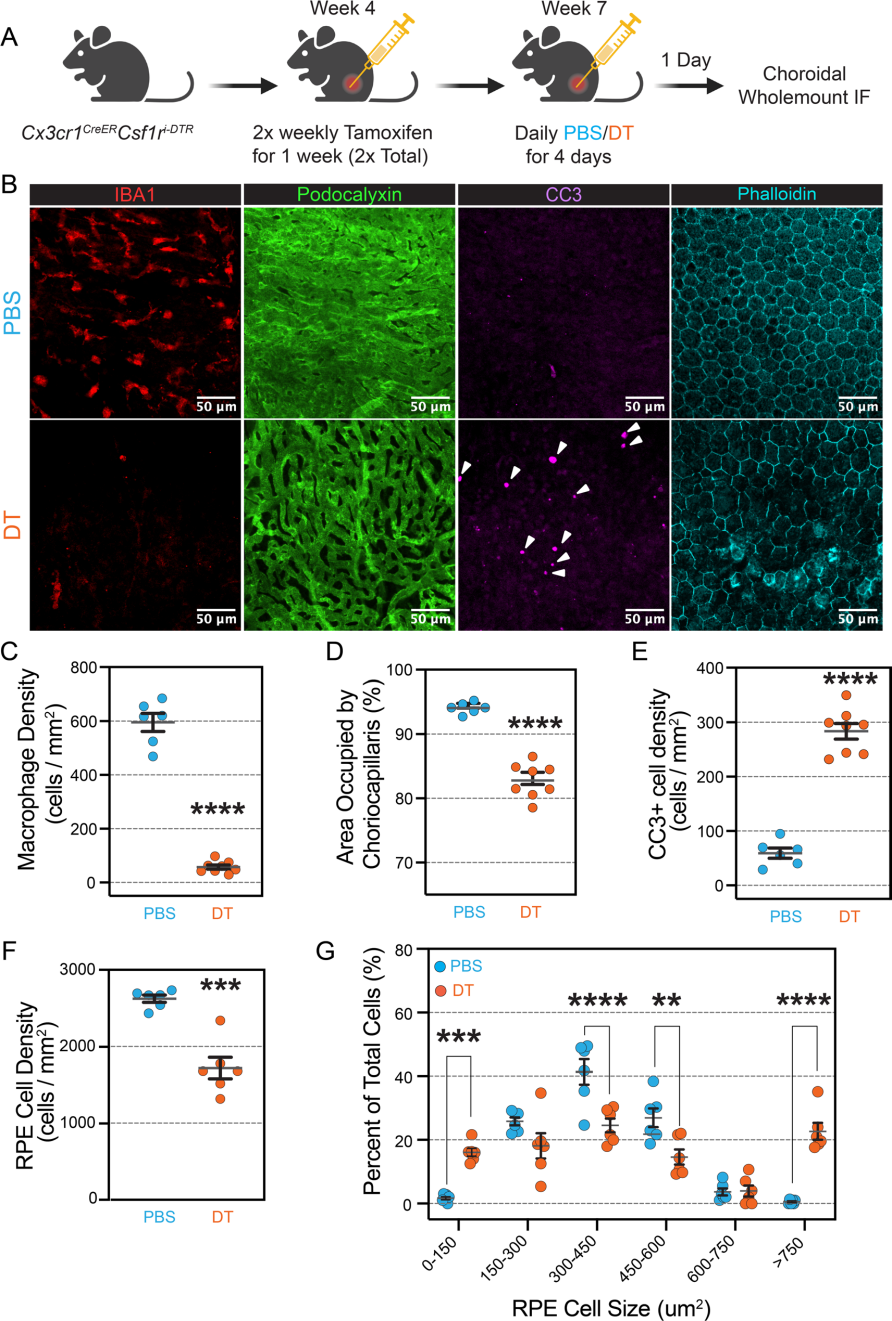
Tissue-resident macrophage ablation reduces CC density and disrupts RPE organization. (**A**) Schematic overview of the injection strategy to deplete tissue-resident macrophages. (**B**) Representative choroidal wholemounts of PBS- and DT-treated mice stained for IBA1 (macrophages), podocalyxin (choriocapillaris), cleaved caspase-3 (CC3; apoptosis marker), and phalloidin (RPE), with white wedges indicating CC3-positive cells. **C-E**. DT treatment reduced macrophage density (Welch’s t-test) and CC density (Welch’s t-test), accompanied by an increase in CC apoptosis (Student’s unpaired t-test, *N* = 6–8 mice per group). **F-G**. Macrophage ablation decreased RPE cell density (Welch’s t-test) and increased heterogeneity of RPE cell size (by two-way ANOVA with Sidak’s multiple comparisons test, *N* = 6 mice per group). ** *p* < 0.01, *** *p* < 0.001, **** *p* < 0.0001

Choroidal macrophages were recently shown to have a dual ontogeny, including both fetal and monocyte-derived populations [26]. To independently investigate tissue-resident macrophages, we treated *Cx3cr1*^*CreER*^*Csf1r*^*i−DTR*^ mice with 2 doses of tamoxifen at 4 weeks of age, followed by a 3-week wash out period to allow circulating monocytes to repopulate prior to DT treatment (Fig. 3A). Mice then received 4 daily IP injections of PBS or DT at 7 weeks of age to deplete tissue-resident macrophages (Fig. 3A). Representative choroidal wholemount images are shown in Fig. 3B. This strategy reduced microglia by 93.8% (*p* < 0.001, Fig S3) and depleted choroidal macrophages by 90.4% (*p* < 0.0001, Fig. 3C). Furthermore, tissue-resident macrophage ablation resulted in a 11.3% absolute reduction in CC density (*p* < 0.0001, Fig. 3D), a 4.8-fold increase in apoptotic CC cells (*p* < 0.0001, Fig. 3E), a 34.5% reduction in RPE density (*p* < 0.001, Fig. 3F), and the distribution of RPE cell size was markedly more heterogeneous (Fig. 3G). These data show that tissue-resident macrophage ablation phenocopies pan-ocular macrophage depletion, indicating that tissue-resident macrophages maintain CC-RPE integrity.

**Fig. 4:**
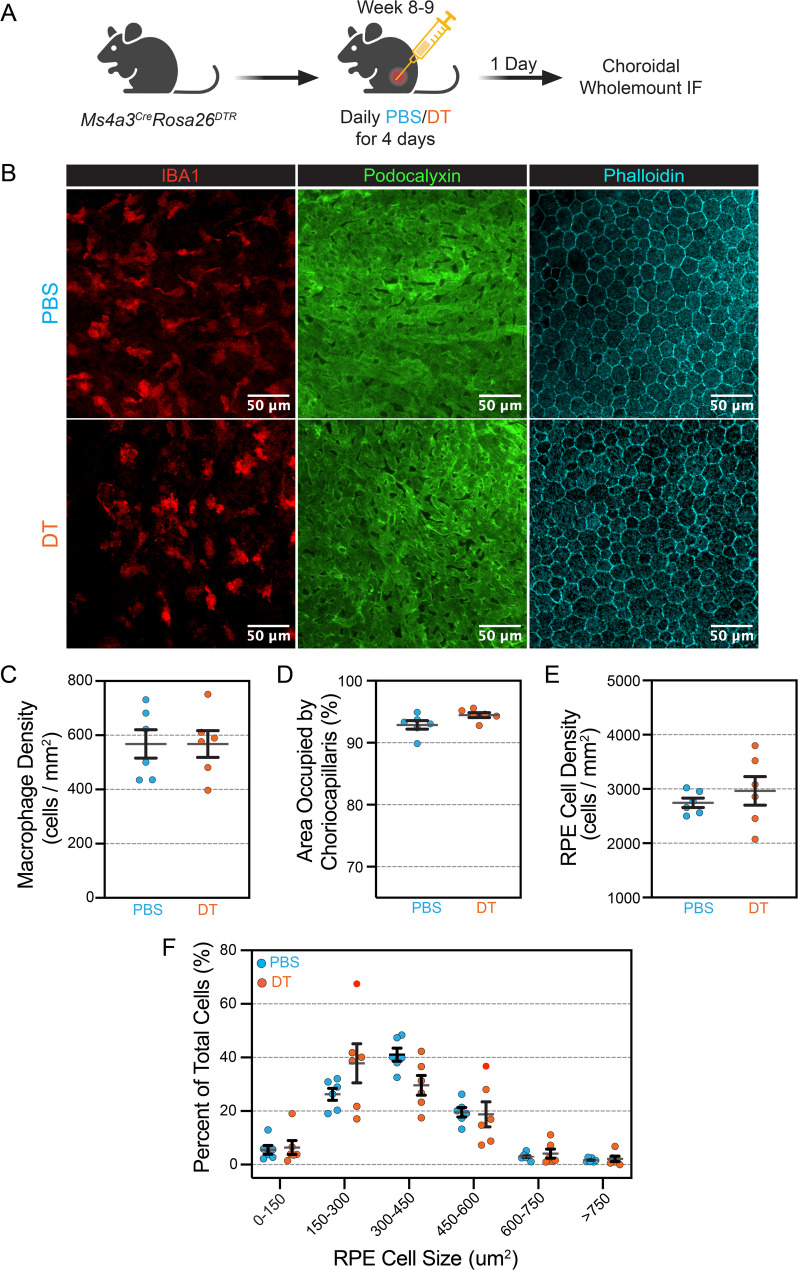
Monocyte-derived macrophages are not necessary for maintenance of CC-RPE homeostasis. (**A**) Schematic overview of the treatment strategy to deplete monocyte-derived macrophages. (**B**) Representative choroidal whole mount images of PBS- and DT-treated *Ms4a3*^*Cre*^*Rosa26*^*DTR*^ mice stained for IBA1 (macrophages), podocalyxin (CC), and phalloidin (RPE). **C-F**. Quantification of choroidal macrophage density (Student’s unpaired t-test, *N* = 6 mice per group), CC density (Student’s unpaired t-test, *N* = 4–5 mice per group), and RPE density (Student’s unpaired t-test) and cell size distribution (two-way ANOVA with Sidak’s multiple comparisons test, *N* = 6 mice per group) revealed no significant differences between DT- and PBS-treated groups

To delineate the contribution of monocyte-derived macrophages to CC-RPE homeostasis, we generated *Ms4a3*^*Cre*^*Rosa26*^*DTR*^ mice. In this model, the *Ms4a3* promoter drives Cre expression in granulocyte-monocyte progenitors, enabling depletion of monocyte-derived macrophages with DT treatment without affecting fetal-derived macrophages [33]. Mice received 4 daily IP injections of PBS or DT at 8–9 weeks of age to ablate monocyte-derived macrophages (Fig. 4A), and representative choroidal wholemount images are shown in Fig. 4B. In contrast to pan-ocular or tissue-resident macrophage depletion, this strategy did not reduce microglia (Fig S4) or choroidal macrophages (Fig. 4C). Further, CC density (Fig. 4D) and RPE density and distribution (Fig. 4E-F) were also unaffected. Additionally, the absence of CC and RPE abnormalities following DT administration indicates that DT itself does not change the morphology of the CC-RPE unit. Collectively, these findings demonstrate that monocyte-derived macrophage depletion fails to phenocopy pan-ocular or tissue-resident macrophage ablation, supporting that tissue-resident macrophages are the primary macrophage population maintaining CC-RPE integrity.

**Fig. 5:**
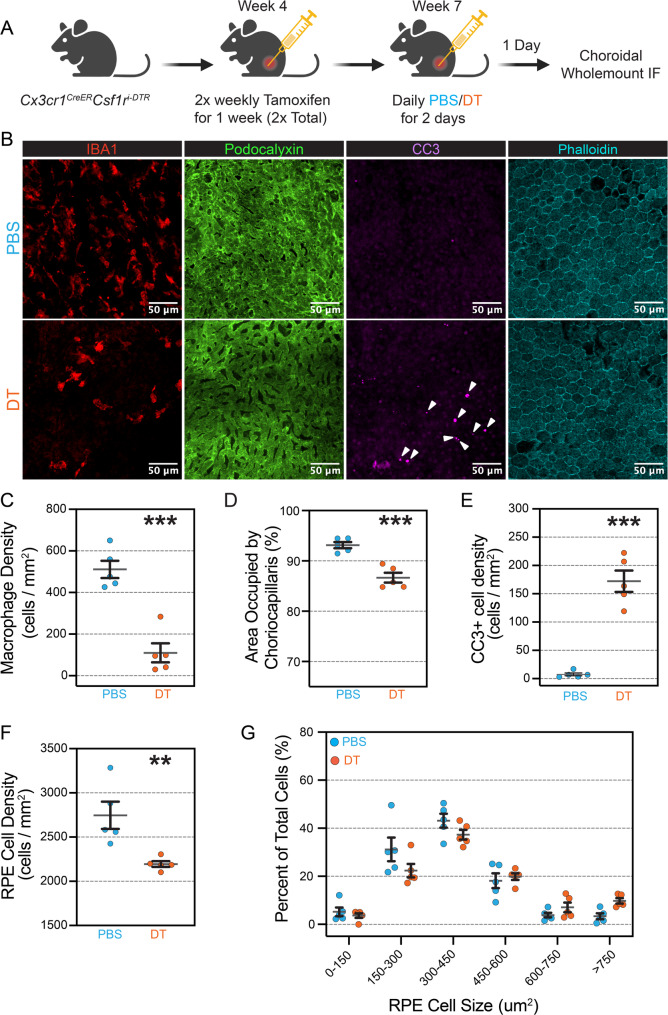
CC and RPE degradation occur simultaneously following macrophage ablation. (**A**) Schematic overview of injection strategy. (**B**) Representative choroidal wholemount images of PBS- and DT-treated mice stained with podocalyxin (CC), IBA1 (macrophages), cleaved caspase-3 (CC3; apoptosis marker), and phalloidin (RPE), with white wedges indicating CC3-positive cells. **C-E.** DT treatment reduced choroidal macrophage and CC density (Student’s unpaired t-test) and increased apoptotic CC cells compared with PBS controls (Welch’s t-test, *N* = 5 mice per group). **F-G.** While RPE cell density was reduced following DT treatment (Student’s unpaired t-test), RPE cell size distribution remained unchanged (two-way ANOVA followed by Sidak’s multiple comparisons test, *N* = 5 mice per group). Statistical analyses: An unpaired two-tailed Student’s *t*-tests was used for macrophage density, CC density, CC3 staining, and RPE density. RPE cell size distributions were analyzed by two-way ANOVA with Šidák’s multiple comparisons test. ** *p* < 0.01, *** *p* < 0.001

Since the CC and RPE function as a homeostatic unit, we sought to investigate whether the CC or RPE phenotype arises first following macrophage ablation. *Cx3cr1*^*CreER*^*Csf1r*^*i−DTR*^ mice were treated with 2 doses of tamoxifen at 4 weeks of age, followed by a 3-week wash out period, identically to Fig. 3. Administration of PBS or DT was limited to 2 daily IP injections at 7 weeks of age to shorten the duration of ablation in order to capture early CC or RPE phenotypes (Fig. 5A). Representative choroidal wholemount images are shown in Fig. 5B. This strategy reduced macrophage density by 78.5% (*p* < 0.001, Fig. 5C), and was sufficient to induce a 6.5% absolute reduction in CC density (*p* < 0.001, Fig. 5D) with a 23.0-fold increase in apoptotic CC cells (*p* < 0.001, Fig. 5E). While RPE density decreased by 20.1% (*p* < 0.01, Fig. 5F), distribution of RPE cell size remained unchanged (Fig. 5G). Despite reduced CC and RPE phenotypes compared to longer depletion strategies, changes occurred simultaneously, indicating that CC and RPE degeneration cannot be uncoupled and reinforcing their interdependent homeostatic relationship.

**Fig. 6:**
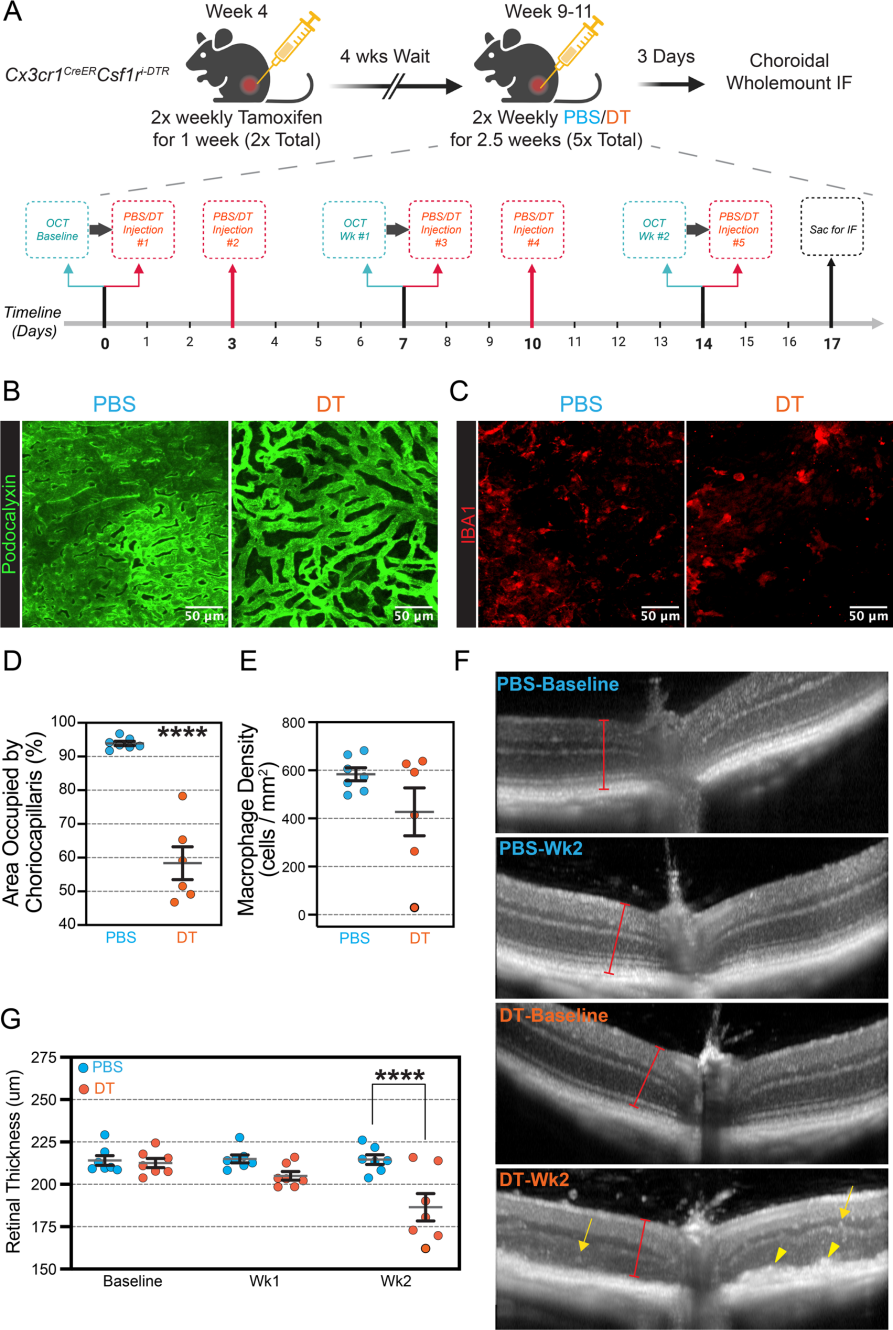
Long-term ablation of choroidal macrophages induces AMD-like features. **A**. Experimental schematic illustrating the treatment paradigm. **B-C**. Representative choroidal wholemount images of podocalyxin (CC) and IBA1 (macrophages). **D**. CC density was significantly reduced by long-term DT treatment (*N* = 6–7 mice per group, Welch’s t-test). **E**. Macrophage density was not changed (*N* = 6–7 mice per group, Welch’s t-test). **F**. Representative OCT images from PBS- and DT-treated mice. Retinal thickness was measured using the red vertical bar at an identical distance from the optic nerve head across conditions and time points. Yellow arrows denote hyperreflective foci. Yellow wedges highlight areas of sub-retinal depoits. **G.** Retinal thickness was significantly reduced at week 2 (*N* = 7 mice per group, two-way ANOVA followed by Sidak’s multiple comparisons test) in DT-treated mice. * *p* < 0.05, **** *p* < 0.0001

Since altered CC-RPE homeostasis is a central pathophysiology of AMD, we investigated the long-term consequences of choroidal macrophage ablation using longitudinal optical coherence tomography (OCT) imaging. *Cx3cr1*^*CreER*^*Csf1r*^*i−DTR*^ mice were treated with 2 tamoxifen doses at 4 weeks of age followed by a 4 week washout period to target long-lived, tissue-resident macrophages (Fig. 6A). Mice then received either PBS or DT twice weekly for 2.5 weeks, for a total of 5 injections (Fig. 6A). Longitudinal OCT imaging was performed at baseline and at weeks 1 and 2 following DT or PBS administration (Fig. 6A). DT treatment resulted in a 35.5% absolute reduction in CC density (*p* < 0.0001, Fig. 6B, D). Alternatively, choroidal macrophages were not significantly different (Fig. 6C, E). However, macrophages in DT-treated mice displayed a rounded morphology distinct from the regular appearance observed in PBS controls, suggesting repopulation by monocyte-derived macrophages with altered morphology and potentially impaired function. DT-treated mice developed progressive retinal abnormalities, including the emergence of hyperreflective foci within the retinal layers (yellow arrows) and subretinal deposits (yellow arrowheads, Fig. 6F). Furthermore, DT treatment decreased retinal thickness by 13.1% (*p* < 0.0001) at week 2 (Fig. 6G). These features are consistent with an AMD-like phenotype.

**Fig. 7:**
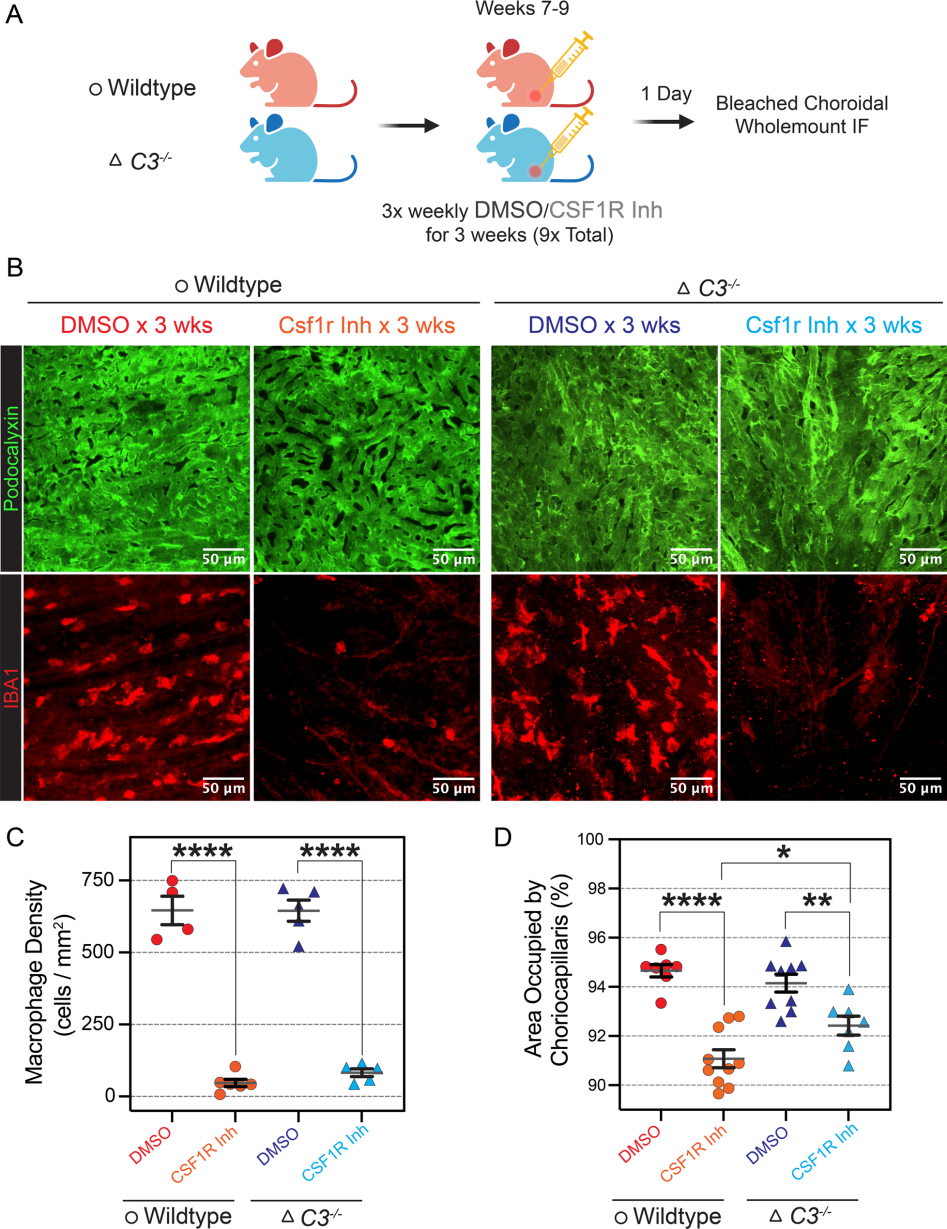
*C3*^*−/−*^ mice exhibit reduced CC degeneration following CSF1R inhibition compared to wildtype mice. (**A**) Experimental schematic illustrating the treatment paradigm. (**B**) Representative immunofluorescence images of choroidal flatmounts stained for IBA1 (macrophages) and podocalyxin (CC). (**C**) Macrophages are comparably reduced in both *C3*^*−/−*^ and wildtype mice (*N* = 4–6 mice per group). (**D**) CSF1R inhibition resulted in partial CC loss in *C3*^*−/−*^ mice (*N* = 7–10 mice per group). One-way ANOVA followed by Sidak’s multiple comparisons test. * *p* < 0.05, ** *p* < 0.01, **** *p* < 0.0001

Since altered CC-RPE homeostasis is a central pathophysiology of AMD and long-lived macrophage ablation causes CC-RPE homeostasis disruption with AMD-like features and MAC accumulation, we investigated if complement signaling was necessary for this phenotype. Wildtype and *C3*^*-/-*^ mice were treated with either vehicle (DMSO) or CSF1R antagonist 3 times per week for 3 weeks to ablate macrophages (Fig. 7A). CSF1R inhibition resulted in a robust and comparable reduction in choroidal macrophage density in both wildtype and *C3*^*-/-*^ mice (Fig. 7B–C), confirming effective macrophage depletion across genotypes. Despite this equivalent loss of macrophages, CC density was differentially affected. Wildtype mice exhibited a 3.6% absolute reduction in CC density following CSF1R inhibition (*p* < 0.0001, Fig. 7D). Alternatively, CC density was decreased by 1.4% in *C3*^*-/-*^ mice (*p* < 0.01, Fig. 7D). While wildtype and *C3*^*-/-*^ mice were not significantly different with DMSO treatment, wildtype CSF1R-treated mice demonstrated a 1.9% absolute reduction compared to CSF1R-treated *C3*^*-/-*^ mice (*p* < 0.05, Fig. 7D). These findings indicate that loss of C3 partially protects against CC-RPE disruption induced by macrophage ablation. However, the incomplete preservation of CC structure in *C3*^*-/-*^ mice suggests that additional, complement-independent mechanisms also contribute to CC-RPE homeostasis.

## Discussion

In this study, we found that pan-ocular macrophage ablation reduced CC density and VEGF levels, altered RPE density and morphology, and increased MAC deposition. Maintenance of the CC-RPE unit is mediated by tissue-resident macrophages, as specific depletion of this population phenocopied pan-ocular macrophage ablation. In contrast, depletion of monocyte-derived macrophages did not alter CC density of RPE organization. Additionally, we observed that the CC-RPE unit is interdependent, as early structural changes in these tissues occurred simultaneously. Tissue-resident choroidal macrophage ablation led to progressive CC and RPE loss over time, mimicking AMD-like pathology. Finally, genetic deletion of C3 partially protected against CC loss following macrophage ablation. Together, these data show that tissue-resident choroidal macrophages are essential for maintaining CC-RPE health and that this process is partially complement-dependent.

Our results agree with and expand upon prior work by Fortmann et al., who demonstrated that depletion of fetal-derived, long-lived FOLR2^+^ macrophages resulted in CC degeneration and AMD-like phenotypes [26]. In their study, LYVE1^+^FOLR2^+^ macrophage ontogeny was assessed using *Ms4a3*^*Cre*^ reporter mice, in which granulocyte-monocyte progenitors are labeled. They found that LYVE1^+^FOLR2^+^ macrophages were negative for *Ms4a3* expression, suggesting a prenatal origin. Further, FOLR2⁺ macrophages were ablated using *Lyve1*^*Cre*^*Csf1r*^*iDTR*^ mice, resulting in CC-RPE homeostasis disruption, identically to our phenotype. We selectively ablated tissue-resident and monocyte-derived macrophages using *Cx3cr1*^*CreER*^*Csf1r*^*i−DTR*^ mice with a tamoxifen pulse-chase and *Ms4a3*^*Cre*^*Rosa26*^*DTR*^ mice, to directly test the function of each population. Although we took distinct approaches, both studies are complementary and found that long-lived, tissue-resident macrophages are the key macrophage population responsible for maintaining CC integrity, while the monocyte-derived macrophage population was dispensable. In addition, we have expanded further on this AMD-like phenotype by identifying the partial mechanism.

In humans, MAC deposition increases in the CC with both aging and AMD [18]. Additionally, choroidal macrophage populations are altered, increasing in number and shifting toward a rounded, activated morphology [21]. Further, AMD patients exhibit elevated peripheral blood monocytes [34], and in mice, the proportion of choroidal macrophages that are monocyte-derived increases with age [26]. Together, these findings suggest that an overabundance of monocyte-derived macrophages, or a relative reduction of long-lived, tissue-resident choroidal macrophages, may contribute to AMD pathology, potentially through complement-dependent mechanisms. Our findings support this hypothesis, as C3 deficiency partially attenuated CC loss following macrophage ablation, indicating that tissue-resident, long-lived choroidal macrophages inhibit complement overactivation to maintain CC-RPE homeostasis. This hypothesis is supported by the fact that a subset of tissue-resident peritoneal macrophages has been shown to exhibit enhanced phagocytosis of C3-opsonized targets dependent on the *Vsig4*/CRIg receptor, which is co-expressed with FOLR2 and LYVE1 in human choroidal macrophages [22, 23, 35]. These data suggest that long-lived, tissue-resident choroidal macrophages may have a specialized capacity to limit excess complement deposition via phagocytosis, thereby preserving CC-RPE integrity. This is further supported by transcriptomic studies of LYVE1^+^FOLR2^+^ choroidal macrophages, which demonstrate enrichment in lipid metabolism pathways [26], likely due to a need for clearance of lipid-rich debris after phagocytosis. Loss of this specialized phagocytic and lipid-metabolism enriched fetal-derived macrophage population could therefore allow unchecked complement activation and progressive CC-RPE degeneration.

We interestingly found that DT treatment in *Cx3cr1*^*CreER*^*Csf1r*^*i−DTR*^ mice reduced serum levels of complement (Fig S2). This could be a contrasting reduction in serum complement by macrophage ablation or a result of tissue complement activation that secondarily causes serum complement consumption. Future studies are needed to unravel whether tissue resident macrophage ablation impacts complement activity in other tissues, its production, or its serum regulation.

Since C3 deficiency was only partially protective, additional mechanisms remain unexplored. Tissue-resident choroidal macrophages may preserve CC integrity by directly supporting RPE function since RPE-CC homeostasis is so tightly intertwined. Tissue-resident cardiac macrophages express insulin-like growth factor-1 (IGF-1), and macrophage-specific IGF-1 deletion impairs tissue growth and organ function [36]. Additionally, macrophage-derived IGF-1 has also been shown to promote VEGF expression and endothelial cell proliferation in the neonatal intestine [37]. Since the IGF-1 receptor is expressed in the RPE [38], and IGF-1 can increase VEGF expression in RPE cells [39, 40], a potential complement-independent mechanism exists where choroidal macrophages express IGF-1, which then signals to the RPE to produce VEGF to maintain the CC. Depletion of tissue-resident macrophages may consequently reduce local IGF-1 availability, decrease RPE-derived VEGF levels, and ultimately drive both RPE and CC degeneration. An additional hypothesis is that reduced VEGF signaling itself may exacerbate complement activation, as VEGF has been shown to regulate expression of local complement inhibitors [41]. Thus, choroidal macrophage depletion may disrupt CC-RPE homeostasis not only through loss of complement control, but also through impairment of RPE function.

Since our data demonstrate that macrophage ablation disrupts both CC and RPE, we sought to determine whether one phenotype preceded, and potentially caused, the other. However, we were unable to temporally separate the two processes as only 2 days of DT treatment induced early CC loss and RPE changes simultaneously. These findings reinforce the concept that the CC and RPE function as a tightly coupled, interdependent homeostatic unit, in which the disruption of either component disrupts the other in a self-reinforcing loop.

There are several limitations to this study. First, the strategy used to deplete tissue-resident macrophages also resulted in depletion of microglia, so it remains unclear whether microglia also contribute to CC-RPE maintenance. Future studies which selectively ablate microglia or choroidal macrophages without microglia alterations may clarify their role. Additionally, the partial CC rescue observed in C3-deficient mice indicates that complement-independent pathways also contribute to CC and RPE degeneration. Follow up studies will explore these alternative mechanisms, including the role of macrophage-derived IGF-1 in supporting the CC-RPE unit. Third, we did not quantify RPE density in C3-deficient mice, so it is unclear how macrophage depletion affects RPE organization during complement deficiency. Finally, we did not establish a direct link between choroidal macrophages and complement. It remains unclear whether complement activation in the choroid is a consequence or cause of CC and RPE damage.

## Conclusions

In summary, we demonstrate that tissue-resident choroidal macrophages are essential for maintaining the CC-RPE homeostatic unit, while monocyte-derived macrophages are dispensable. We identified that tissue-resident macrophages maintain CC-RPE homeostasis by complement dependent and independent mechanisms, and provide insight into how their loss drives CC-RPE degeneration and AMD-like pathology.

## Supplementary Information

Supplementary Material 1. Fig S1. Title of Data: Isotype IgG Controls. Description of Data: Isotype IgG controls for rabbit, rat, and goat staining with simultaneous positive staining in other channels, indicating specificity.

Supplementary Material 2. Fig S2. Title of Data: Serum ELISA. Description of Data: Serum ELISA measurements show that DT treatment reduces systemic complement activity in Cx3cr1^CreER^Csf1r^i-DTR^ mice. N=6 mice per group, Student’s unpaired t-test, ** *p*<0.01.

Supplementary Material 3. Fig S3. Title of Data: DT treatment of Cx3cr1^CreER^Csf1r^i-DTR^ mice reduces microglia density. Description of Data A. Schematic overview of the injection strategy B. Representative retinal wholemount images of the deep capillary plexus in PBS- and DT-treated mice stained with CD31 (endothelial cells) and IBA1 (macrophages). C. DT treatment significantly reduced retinal microglia density (N=7-8 mice per group, Mann–Whitney test, *** *p* < 0.001)

Supplementary Material 4. Fig S4. Title of Data: Retinal microglial density remains unchanged following depletion of monocyte-derived macrophages. Description of Data: A. Schematic overview of the treatment strategy to deplete monocyte-derived macrophages. B. Representative retinal wholemount images of the deep capillary plexus in PBS- and DT-treated Ms4a3^Cre^Rosa26^DTR^ mice stained with CD31 (endothelial cells) and IBA1 (macrophages). C. DT treatment had no significant effect on retinal microglial density (N=6 mice per group, Student’s unpaired t-test)

## Data Availability

All data will be available upon reasonable request to the corresponding author.

## Abbreviations

AMD: Age-related Macular Degeneration
RPE: Retinal Pigment Epithelium
CNV: Choroidal Neovascularization
CC: Choriocapillaris
VEGF: Vascular Endothelial Growth Factor
VEGFR2: Vascular Endothelial Growth Factor Receptor 2
C3: Complement Component 3
C5: Complement Component 5
MAC: Membrane Attack Complex
CSF1R: Colony-Stimulating Factor 1 Receptor
DT: Diphtheria Toxin
DTR: Diphtheria Toxin Receptor
OCT: Optical Coherence Tomography
ANOVA: Analysis of variance
TBS: Tris-buffered saline solution
TBS-T: Tris-buffered saline solution – tween 20
IF: Immunofluorescence
IP: Intraperitoneal
PBS: Phosphate-buffered saline solution
DMSO: Dimtheyl sulfoxide
IGF-1: Insulin-like growth factor-1
