# Supplementary figures and images for: Tissue-resident macrophages maintain choroidal homeostasis by complement dependent and independent mechanisms
Source: J Neuroinflammation. 2026 May 18;23:241. doi: 10.1186/s12974-026-03872-6 (PMC13352743; doi:10.1186/s12974-026-03872-6)

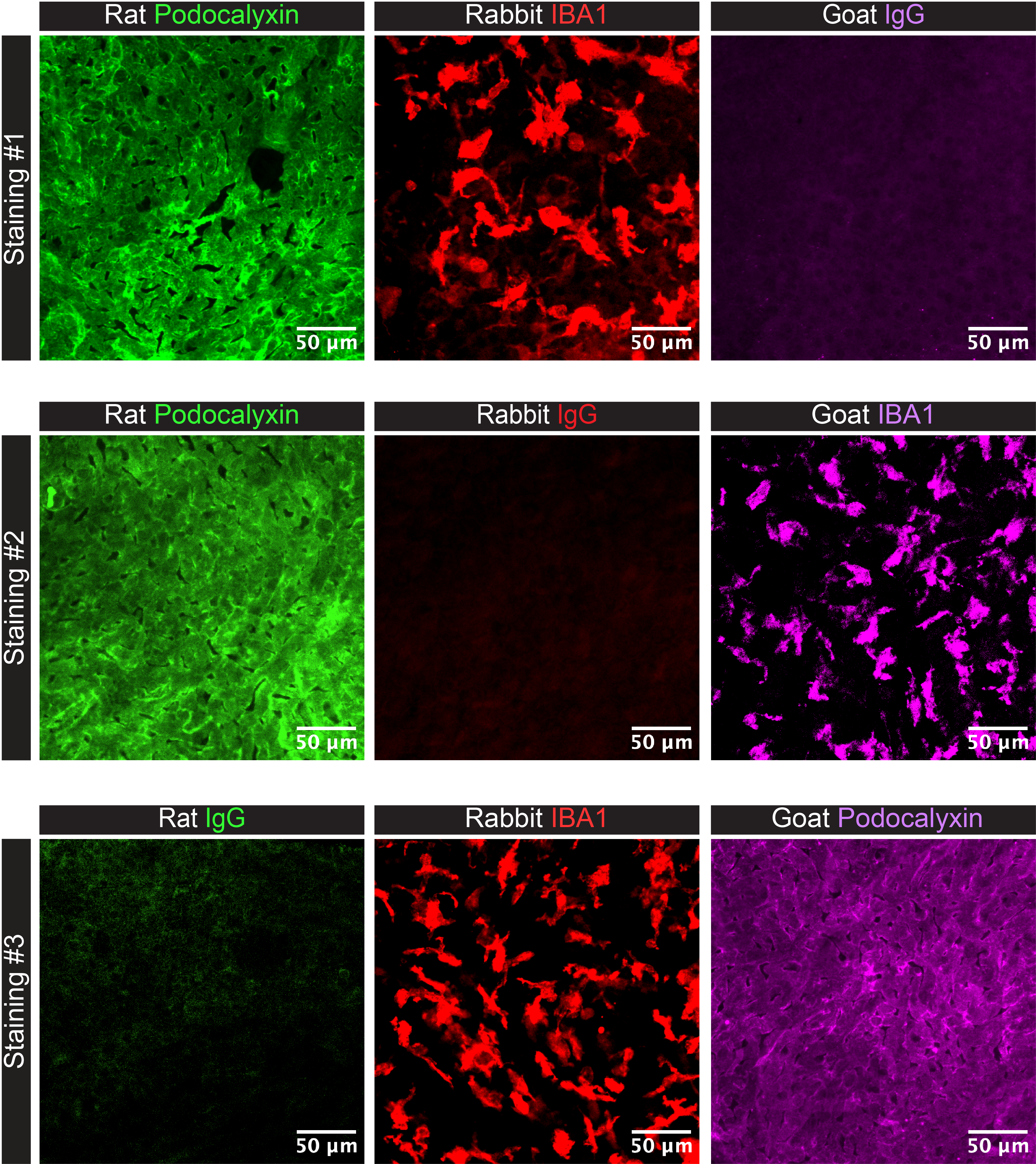

Supplement: Supplementary file 1 — Supplementary Material 1. Fig S1. Title of Data: Isotype IgG Controls. Description of Data: Isotype IgG controls for rabbit, rat, and goat staining with simultaneous positive staining in other channels, indicating specificity. [file 12974_2026_3872_MOESM1_ESM.png]

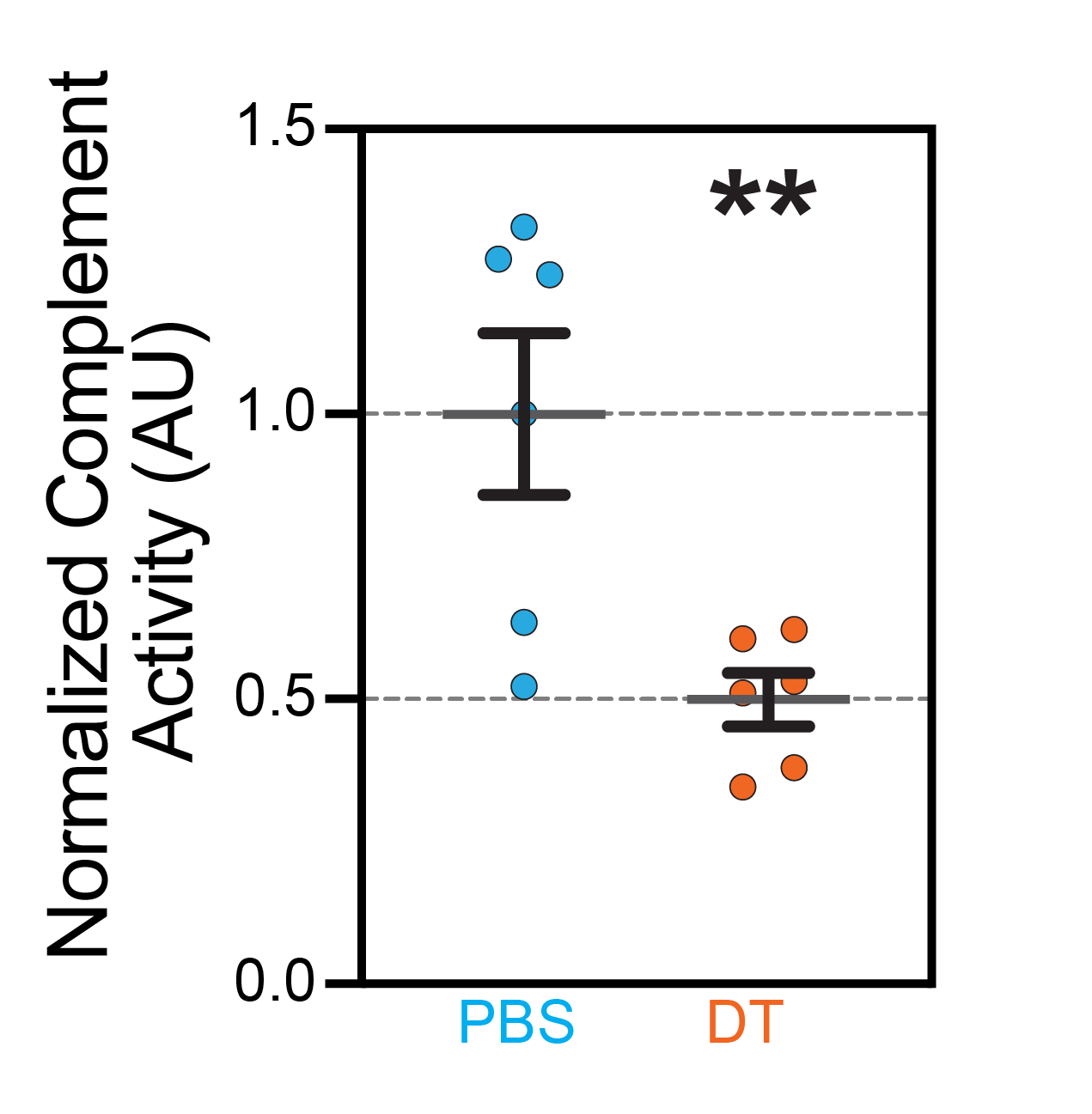

Supplement: Supplementary file 2 — Supplementary Material 2. Fig S2. Title of Data: Serum ELISA. Description of Data: Serum ELISA measurements show that DT treatment reduces systemic complement activity in Cx3cr1CreERCsf1ri-DTR mice. N=6 mice per group, Student’s unpaired t-test, ** p<0.01. [file 12974_2026_3872_MOESM2_ESM.png]

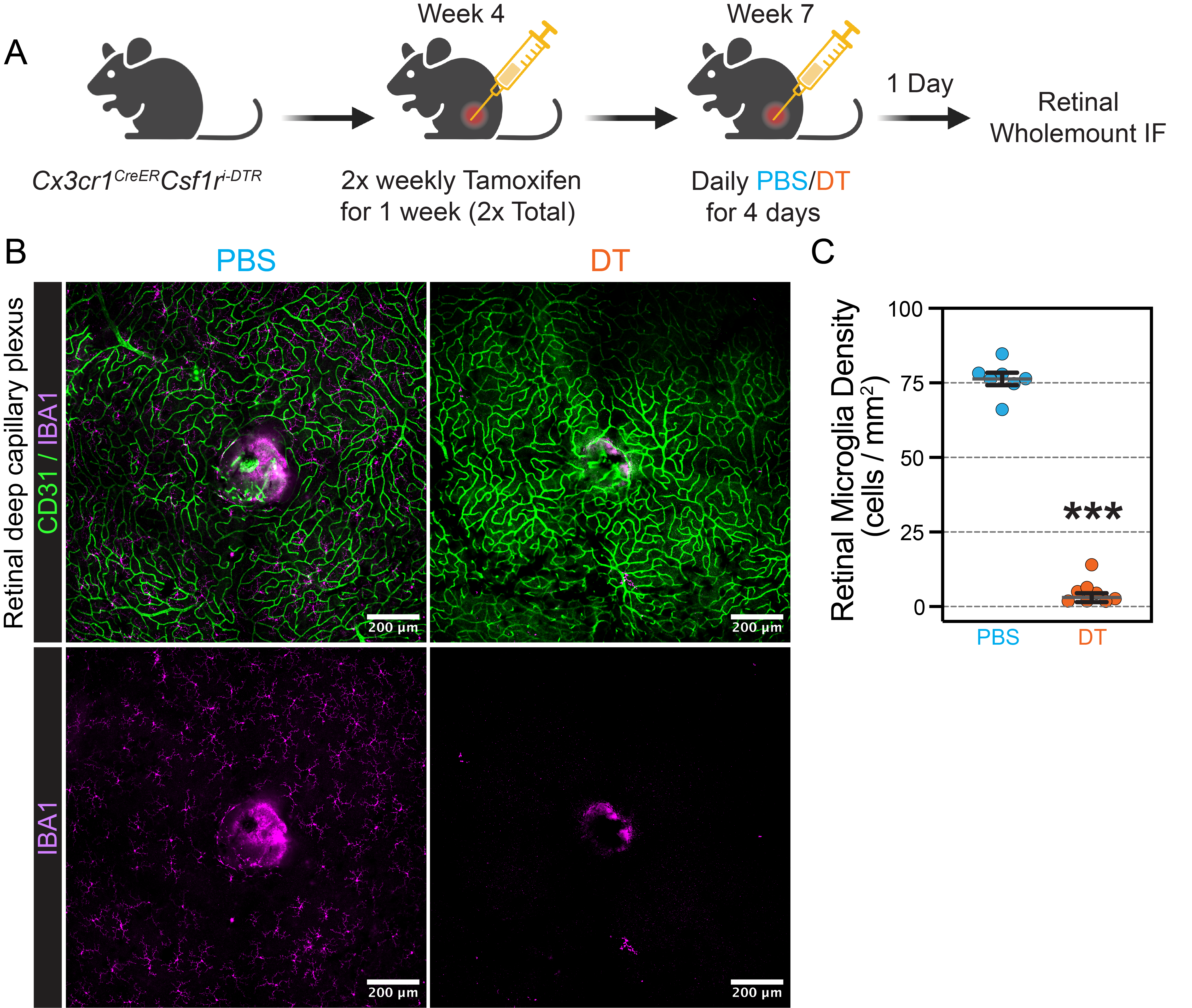

Supplement: Supplementary file 3 — Supplementary Material 3. Fig S3. Title of Data: DT treatment of Cx3cr1CreERCsf1ri-DTR mice reduces microglia density. Description of Data A. Schematic overview of the injection strategy B. Representative retinal wholemount images of the deep capillary plexus in PBS- and DT-treated mice stained with CD31 (endothelial cells) and IBA1 (macrophages). C. DT treatment significantly reduced retinal microglia density (N=7-8 mice per group, Mann–Whitney test, *** p < 0.001) [file 12974_2026_3872_MOESM3_ESM.png]

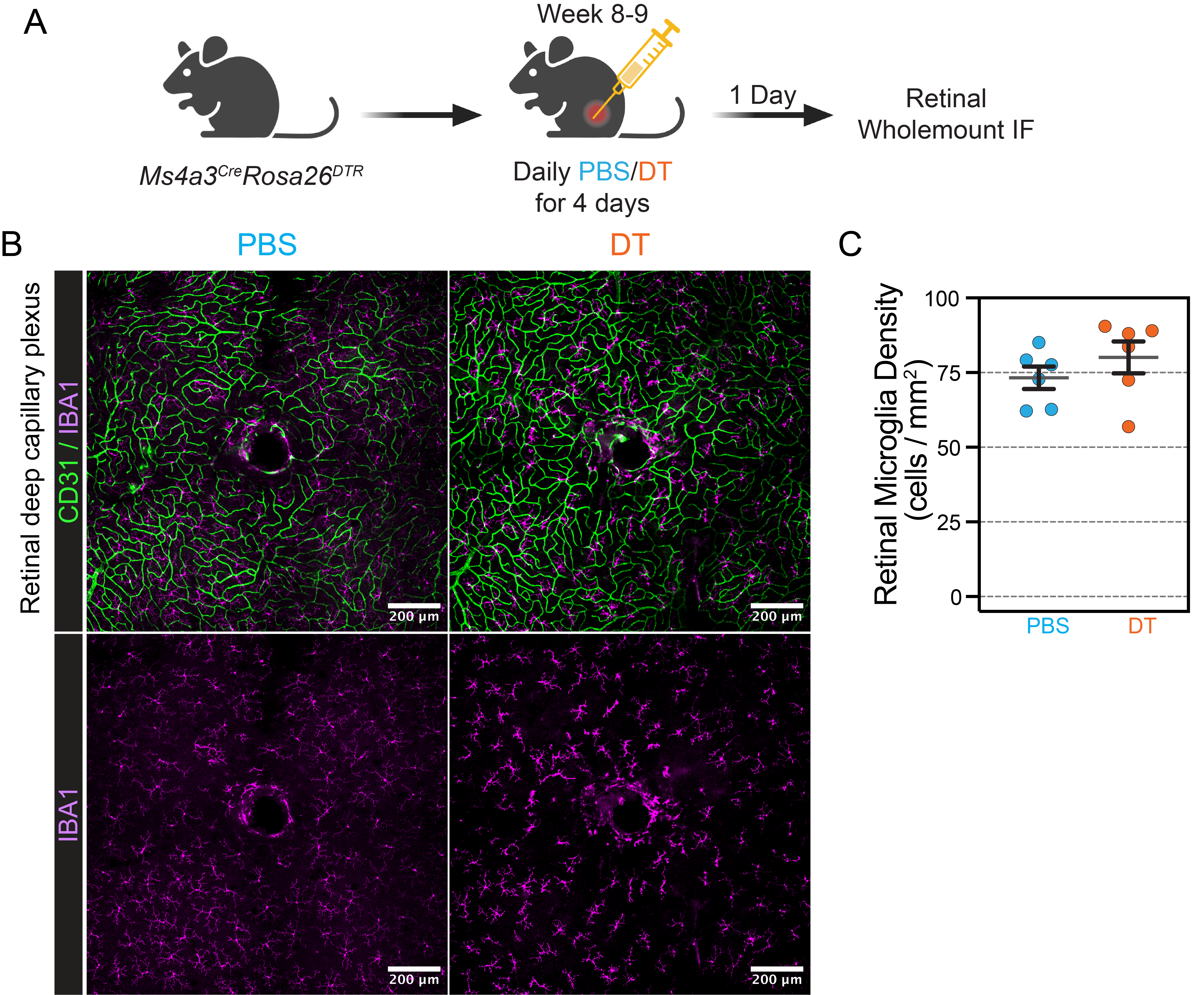

Supplement: Supplementary file 4 — Supplementary Material 4. Fig S4. Title of Data: Retinal microglial density remains unchanged following depletion of monocyte-derived macrophages. Description of Data: A. Schematic overview of the treatment strategy to deplete monocyte-derived macrophages. B. Representative retinal wholemount images of the deep capillary plexus in PBS- and DT-treated Ms4a3CreRosa26DTR mice stained with CD31 (endothelial cells) and IBA1 (macrophages). C. DT treatment had no significant effect on retinal microglial density (N=6 mice per group, Student’s unpaired t-test) [file 12974_2026_3872_MOESM4_ESM.png]
